# Phosphorylation of the *Bacillus subtilis* Replication Controller YabA Plays a Role in Regulation of Sporulation and Biofilm Formation

**DOI:** 10.3389/fmicb.2018.00486

**Published:** 2018-03-21

**Authors:** Tránsito García García, Magali Ventroux, Abderahmane Derouiche, Vladimir Bidnenko, Sara Correia Santos, Céline Henry, Ivan Mijakovic, Marie-Françoise Noirot-Gros, Sandrine Poncet

**Affiliations:** ^1^Micalis Institute, INRA, AgroParisTech, Université Paris-Saclay, Jouy-en-Josas, France; ^2^Systems and Synthetic Biology, Chalmers University of Technology, Göteborg, Sweden; ^3^Argonne National Laboratory, Biosciences Division, Argonne, IL, United States

**Keywords:** *Bacillus subtilis*, Ser/Thr kinase, sporulation, biofilm, replication initiation control

## Abstract

*Bacillus subtilis* cells can adopt different life-styles in response to various environmental cues, including planktonic cells during vegetative growth, sessile cells during biofilm formation and sporulation. While switching life-styles, bacteria must coordinate the progression of their cell cycle with their physiological status. Our current understanding of the regulatory pathways controlling the decision-making processes and triggering developmental switches highlights a key role of protein phosphorylation. The regulatory mechanisms that integrate the bacterial chromosome replication status with sporulation involve checkpoint proteins that target the replication initiator DnaA or the kinase phosphorelay controlling the master regulator Spo0A. *B. subtilis* YabA is known to interact with DnaA to prevent over-initiation of replication during vegetative growth. Here, we report that YabA is phosphorylated by YabT, a Ser/Thr kinase expressed during sporulation and biofilm formation. The phosphorylation of YabA has no effect on replication initiation control but hyper-phosphorylation of YabA leads to an increase in sporulation efficiency and a strong inhibition of biofilm formation. We also provide evidence that YabA phosphorylation affects the level of Spo0A-P in cells. These results indicate that YabA is a multifunctional protein with a dual role in regulating replication initiation and life-style switching, thereby providing a potential mechanism for cross-talk and coordination of cellular processes during adaptation to environmental change.

## Introduction

Bacteria developed various regulatory strategies to ensure only one initiation event per chromosome per cell cycle and to coordinate chromosome replication with cell division. The Gram-positive bacterium *Bacillus subtilis* is a developmental model organism capable of differentiating into spores or forming biofilms in response to environmental signals. How *B. subtilis* couples the developmental decision-making with the replication status is not fully understood. In *B. subtilis*, multiple regulatory processes contribute to regulating DNA replication at the onset of sporulation (Veening et al., 2009). Essentially, these mechanisms target the master initiator protein DnaA and modulate its activity (Zakrzewska-Czerwinska et al., 2007; Katayama et al., 2010; Skarstad and Katayama, 2013). During vegetative growth, DnaA binds to the replication origin *oriC* and forms high-order oligomeric assemblies leading to unwinding of the duplex DNA for subsequent loading of the replication machinery. Several anti-cooperativity factors regulate DnaA oligomerization at *oriC* during the different life-styles of the bacterium (Jameson and Wilkinson, 2017). During vegetative growth, three regulatory proteins, YabA, DnaD, and Soj, down-regulate replication initiation by binding DnaA to prevent its cooperative binding at origin sequences (Merrikh and Grossman, 2011; Bonilla and Grossman, 2012; Scholefield and Murray, 2013). The initiation controller YabA also exerts its regulatory control as part of a multimeric complex with DnaA and DnaN, associated with the replication fork during most of the replication cycle (Noirot-Gros et al., 2002, 2006; Hayashi et al., 2005; Cho et al., 2008; Soufo et al., 2008; Felicori et al., 2016). YabA is a small Zn-binding protein with an overall tri-dimensional structure composed of four N-terminal helix bundle in a tetramer connected to four monomeric C-terminal domains by flexible linkers (Felicori et al., 2016). Its unique structure defines YabA as a hub-like protein, able to interact with several partners to control and coordinate replication initiation with other cellular processes (Noirot-Gros et al., 2006; Felicori et al., 2016). In cells engaged in sporulation, the SirA protein acts, similarly to Soj, by inhibiting DnaA loading at replication origin to prevent new rounds of replication initiation (Rahn-Lee et al., 2011; Jameson et al., 2014; Duan et al., 2016).

In *B. subtilis*, the decision to enter the differential states of sporulation or biofilm involves the phosphorylation of a key regulator, Spo0A. The intra-cellular concentration of Spo0A-P is the determining factor that drives the expression of several gene clusters which are necessary for sporulation or production of the biofilm matrix. A well-characterized mechanism for coupling the initiation of sporulation with the DNA replication involves the protein Sda that promotes the reduction of Spo0A-P through inhibition of the activity of the phosphorelay kinase KinA (Burkholder et al., 2001; Veening et al., 2009; Hoover et al., 2010). During vegetative growth, the expression of the *sda* gene is positively regulated by DnaA which also acts as a transcriptional regulator of many genes involved in replication stress and sporulation (Burkholder et al., 2001; Breier and Grossman, 2009). Recent studies established that most of the DnaA transcriptional network is indirectly shaped by Sda, through the DnaA-mediated activation of the *sda* gene expression (Smith and Grossman, 2015; Seid et al., 2017; Washington et al., 2017).

In eukaryotes, the interplay between the cell cycle and the other cellular processes mostly involves the post-translational modifications (PTMs) of proteins by Ser/Thr/Tyr Hanks-type kinases (Hanks et al., 1988; Stancik et al., 2017). The phosphorylation of proteins plays a key role in regulating many aspects of the cell cycle by integrating environmental and cellular signals (Endicott et al., 2012). In eukaryotic cells, DNA replication stress triggers coordinated responses involving signal transduction cascades, leading to the phosphorylation of specific proteins involved in DNA damage checkpoint and processing (Subramanian and Hochwagen, 2014).

The phosphorylation of proteins involved in DNA replication and repair has been identified in various bacteria (Shi et al., 2014; Garcia-Garcia et al., 2016). In *B. subtilis*, the phosphorylation of the single-strand DNA binding proteins SsbA on a tyrosine residue was found to modulate its binding to DNA (Mijakovic et al., 2006), while the replicative helicase DnaC was identified as a substrate of the Hanks-type kinase PrkD (Shi et al., 2014). In *B. subtilis*, phosphorylation at serine or threonine residues is catalyzed by the three Hanks-type Ser/Thr kinases PrkC, PrkD, and YabT. YabT is a transmembrane kinase devoid of the classical extracellular signal receptor domain but containing a DNA-binding motif essential to its activation (Bidnenko et al., 2013). This particular Ser/Thr kinase is mainly expressed at early stage of sporulation as well as in biofilms (Nicolas et al., 2012). Notably, YabT was found to phosphorylate the general DNA recombinase RecA to enforce chromosome integrity during spore development (Bidnenko et al., 2013). YabT also plays a role in down regulation of protein synthesis by phosphorylation of the elongation factor EF-Tu (Pereira et al., 2015). Growing evidence in *B. subtilis* thus points at the involvement of different classes of kinases, kinase activators and substrates, and phosphatases in the regulation of DNA-mediated processes such as replication and repair as well as other processes related to cell cycle and development (Bidnenko et al., 2013; Garcia-Garcia et al., 2016). We purposely investigated whether proteins involved in replication initiation control in *B. subtilis* could be phosphorylated *in vitro* by Ser/Thr and Tyr-kinases. We found that the replication initiation controller YabA was specifically phosphorylated by the developmental Ser/Tr kinase YabT, which is specifically expressed at early stage of sporulation as well as during biofilm formation (Nicolas et al., 2012). The phosphorylated residue was identified by biochemical and genetic approaches. We evidenced that YabA phosphorylation by YabT did not affect replication initiation control. However, our functional analysis hinted at a regulatory role of YabA phosphorylation during sporulation and biofilm formation mediated by Spo0A. These results highlight the multi-functional nature of YabA and its potential role in integrating physiological signals to connect and coordinate chromosomal replication initiation control with cell development.

## Materials and Methods

### Bacterial Strains and Growth Conditions

*Bacillus subtilis* strains used in this work are listed in Supplementary Table S1, PCR primers are listed in Supplementary Table S2, and the plasmids constructs in Supplementary Table S3. All *B. subtilis* strains constructed in this study are derived from the 168 (trp-) derivative CCBS185, containing a neomycin resistance gene under the control of the Lambda Pr promoter (λPr-neo) inserted into *upp* gene. The yabA-T71A and yabA-T71D mutants were constructed at locus by an improved mutation delivery approach as follows. The primers pair yabA fwd + YabAT71AR and YabAT71AF + yabA rev were used to amplify two partially overlapping DNA fragments containing parts of *yabA* gene and the flanking regions. Primers pair yabA fwd + YabAT71DR and YabAT71DF + yabA rev were used for yabA-T71D. The insertion cassette encoding the Lambda CI repressor and the phleomycin-resistance gene was flanked by mutagenized yabA fragments in direct orientation by joining PCR using primers yabA fwd and yabA rev. The amplified fragment was used to transform CCBS185 cells with selection on phleomycin. The obtained clones were controlled for the loss of neomycin resistance due to CI, which inhibits the transcription from the Pr promoter. Finally, homologous regions allow a recombination process leading to the excision of the cassette and cells were isolated by counter-selection for neomycin-resistance. CCBS185 cells were transformed with the chromosome from the strain BMR26 (Bidnenko et al., 2013) to obtain the *yabT* deletion marked with the spectinomycin resistance in CCBS185 background.

Cells were routinely grown in LB medium containing, when needed, spectinomycin 60 μg ml^-1^-1, phleomycin 2 μg ml^-1^, neomycin 5 μg ml^-1^, chloramphenicol 7 μg ml^-1^, or ampicillin 100 μg ml^-1^.

### Protein Purification

The N-terminal 6xHis-tagged YabT protein (cytosolic part containing the kinase domain) was synthesized in the chaperone overproducing strain *Escherichia coli* M15 carrying a pQE-30 derived vector (Jers et al., 2011). Culture was grown under shaking at 37°C to OD600 0.5, induced with 0.1 mM IPTG and grown for an additional 2 h. Cells were then disrupted by sonication and 6xHis-tagged protein was purified on Ni-NTA columns (Qiagen) according to manufacturer’s instructions, desalted on PD-10 columns (GE-Healthcare) and stored in a buffer containing 50 mM Tris-Cl pH 7.5, 100 mM NaCl and 10% glycerol. Protein concentration was estimated using the Bradford assay (Bio-Rad) with BSA as standard.

For YabA expression and purification, the *yabA* gene and derivative genes carrying mutations T71A and T71D were amplified from pGBDU-yabA, pGBDU-yabA-T71A, pGBDU-yabA-T71D bait plasmids with yabA-NdeI and yabA-XhoI primers sets and cloned into NdeI and XhoI sites of pSMG201, which allow cloning of *orf* under the control of the T7 promoter. To purify the proteins, *E. coli* strain ER2566 was transformed with the resultant plasmids. Cells were cultured in LB medium supplemented with 30 μg ml^-1^ kanamycin. When the OD600 reached 0.6, IPTG was added to 0.5 mM to induce expression of the protein and the culture was incubated overnight at 18°C. All subsequent steps were carried out at 4°C. Cells were pelleted and resuspended in buffer A (50 mM Tris pH 8, 1 M NaCl) and were disrupted by sonication and finally centrifuged at 40.000 rpm for 1 h. Proteins from the supernatant were precipitated by adding ammonium sulfate at a final concentration of 20%. After centrifugation, the pellet containing YabA protein was collected and resuspended in buffer B (50 mM Tris pH 8, 0.5 M NaCl). The soluble fraction was concentrated and injected onto a Superdex 200 10/300 GL gel filtration column (GE Healthcare) equilibrated in buffer C (50 mM Tris pH 8, 400 mM NaCl). Fractions containing YabA, YabA-T71A or YabA-T71D were pooled, dialyzed and stored at -20°C in the presence of 50% glycerol (Supplementary Figure S1).

### Identification of the Phosphorylated Residue by Mass Spectrometry

*In vitro* phosphorylation reaction of YabA was carried out in the presence of YabT essentially as described below, with the only difference of using non-radioactive ATP. A typical 30 μL reaction contained 40 μM of YabA, 0.8 μM of YabT, 5 mM MgCl2 and 50 mM Tris–HCl pH 7.4 and 50 μM ATP. After SDS-PAGE, in-gel digestion was performed as described previously by addition of modified trypsin (Promega) dissolved in 50 mM NH4CO3. The resulting peptides were extracted successively with 2% trifluoroacetic acid (TFA) and 50% acetonitrile (ACN) and then with ACN. Vacuum-dried peptide extracts were resuspended in 30 μl of 0.05% TFA, 0.05% HCOOH, and 2% ACN.

A Q Exactive (Thermo Fisher Scientific) coupled to Eksigent nano LC (AB Sciex) was used for the nano-LC-MS/MS analysis. 4 μl were injected on the NanoLC-Ultra system (Eksigent) chain. Sample was loaded at 7.5 μl/min on the precolumn cartridge (PepMap 100 C18, 5 μm, 120 Å, 5 mm Dionex) and desalted with 0.1% HCOOH. Then, peptides were separated with a gradient of acetonitrile on the reverse phase column (C18 Biosphere, 3 μm, 120 Å, 75 μm i.d., 15 cm,). Eluted peptides were analyzed on-line with a Q-Exactive mass spectrometer (Thermo Electron) using a nanoelectrospray interface. Ionization (1.5 kV ionization potential) was performed with stainless steel emitters (30 μm i.d.; Thermo Electron). Peptide ions were analyzed using Xcalibur 2.1 with the following data-dependent acquisition steps: (1) full MS scan [mass-to-charge ratio (m/z) 400–1400] and (2) MS/MS. Step 2 was repeated for the 8 major ions detected in step 1. Dynamic exclusion was set to 40 s. Lock mass option was chosen “best”, MS resolution 70000 at m/z 400, auto gain control was 3e6, maximum injection time 250 ms. For MS2, the resolution was 17500 at m/z 400, auto gain control was 2e5, maximum injection time 120 ms, isolation window m/z = 3, normalized collision energy: 25, underfill ratio 0.5%, intensity threshold 2.5 e4. Charge state: 2.3.4. Dynamic exclusion 40 s.

### Data Processing and Phosphopeptide Validation

A database search was performed with XTandem (version 2011.12.01.1)^1^ and the *B. subtilis* strain 168 database was downloaded from UniProt Database site^2^ (version 2012, 4195 protein entries). This database was merged and in conjunction with reverse and contaminant databases, were searched by Xtandem CYCLONE (version 2011.12.01.1, see foot note 1) using XTandem pipeline (version 3.3.0) developed by PAPPSO platform^3^. Enzymatic cleavage was declared as a trypsin digestion with one possible miss-cleavage. Cys carboxyamidomethylation and Met oxidation were set to static and possible modifications, respectively. Precursor mass was 10 ppm and fragment mass tolerance was 0.02 Th. A refinement search was added with similar parameters except that semi-tryptic peptides, possible N-ter protein acetylation and phosphorylation of serine threonine or tyrosine were searched for the phosphoproteins.

For data of proteomic, only peptides with an *E*-value smaller than 0.1 were reported. Identified proteins were filtered and grouped using XTandem Pipeline^4^ according to: (1) A minimum of two different peptides was required with an *E*-value smaller than 0.05, (2) a protein *E*-value (calculated as the product of unique peptide *E*-values) smaller than 10–4. To take redundancy into account, proteins with at least one peptide in common were grouped. This allowed to group proteins of similar function. Within each group, proteins with at least one specific peptide relatively to other members of the group were reported as sub-groups. For phosphoproteomic data, only one peptide is required with an *E*-value smaller than 0.01 and the protein *E*-value (calculated as the product of unique peptide *E*-values) smaller than 10–2. Identified phosphopeptides were filtered and grouped using XTandem Pipeline confirmed with MaxQuant^5^ and manually validated.

### *In Vitro* Phosphorylation Assay

Phosphorylation reactions were performed in a total volume of 30 μl, in the presence of 40 μM of YabA, YabA-T71A or YabA-T71D, and 1.6 μM of YabT. In addition to the appropriate proteins, the reaction mixture contained 50 μM [γ-32P] ATP (20 μCi/mmol), 5 mM MgCl2, and 50 mM Tris–HCl pH 7.4. Reactions were incubated at 37°C for 1.5 h and stopped by adding loading buffer for SDS-PAGE. Proteins were separated by electrophoresis on denaturing 15% polyacrylamide gels. After drying the gels, radioactive signals were visualized using STORM phosphorimager.

### Isolation of Chromosomal DNA and Q-PCR

DNA was isolated from cultures at exponential phase (OD = 0.3–0.4) in order to determinate the Ori/Ter ratio by Q-PCR. 2 ml of culture was centrifuged and rinced with 10 mM Tris–HCl (pH 8), 10 mM EDTA (pH 8), and 300 mM NaCl. The pellet was resuspended in 200 μL Lysis buffer (50 mM Tris–HCl, 10 mM EDTA, 100 mM NaCl, and 10 mg μgml^-1^ lysozyme). After incubation for 10 min at 37°C, 10 μL sarkozyl 30 % were added to complete cell lysis. The samples were incubated at 65°C for 20 min followed by lithium chloride precipitation to precipitate proteins and other contaminants by salting out. 0.8 volume of 2-propanol was added before centrifugation at 16000 rpm. The pellet was then resuspended in 200 μL double-distilled H_2_O (ddH2O) and 200 μL of 10 M LiCl4. Samples were incubated at -20°C for 20 min and the DNA was precipitated by addition of ethanol and sodium acetate. Precipitated DNA was resuspended in 50 μL of ddH2O. Q-PCRs were carried out in triplicates of 25 μl each and using three dilutions of chromosomal DNA (1, 0.1, and 0.01 pg μL^-1^). 5 μL diluted DNA was used as template and added to a mixture of 12.5 μL ABsolute Blue qPCR SYBR^®^Green ROX Mix (Thermo Scientific) and 0.3 μL primers mix (3 μM each forward and reverse) in a 96-well PCR plates. Primers pair for Origin proximal sequence (OriL3F and OriL3R) and Terminus proximal sequence (TerL3R and TerL3R, Supplementary Table S2) were used in separated reactions and tested for an efficiency greater than or equal to 90%. Reactions were carried out with a Mastercycler^®^ep realplex (Eppendorf) with the following program: 15 min at 95°C, (15 s at 95°C, 1 min at 60°C) ×40 cycles, 15 s at 95°C, 15 s at 60°C, 20 min to reach 95°C, 15 s at 95°C. Results were analyzed with Realplex program (Eppendorf) and quantified by ΔΔCt method.

### Yeast Two-Hybrid Assays

Full length YabA and derivatives YabA-T71A and YabA-T71D, full length DnaA and DnaN proteins were expressed as fusions to the GAL4 binding domain BD or activating domain AD from the vectors pGBDU-C1 and pGAD-C1, respectively. pGBDU and pGAD derivative constructs were introduced by transformation into PJ694-(α) and (a) haploid strains, respectively. Binary interactions were tested by combinational mating of the strains expressing the BD and AD fusions as previously described (Noirot-Gros et al., 2006). Interacting phenotypes were tested by the ability of the diploid cells to grow on selective media SD-LUH and SD-LUA.

### Construction of the GFP Fusions and Epifluorescence Microscopy

The N-terminal tagged GFP-YabA proteins were conditionally expressed from the xylose-inducible promoter Pxyl, at the ectopic *amyE* locus in the *B. subtilis* chromosome. Primers yabA-apa and yabA-R-salI, carrying ApaI and SalI restriction sites, respectively, were used to PCR amplify the wild-type and mutant *yabA* genes from the corresponding bait vectors. The PCR fragments and pSG1729 were digested with ApaI + SalI. *E. coli* DH10B cells were transformed by the ligation mixtures, and single transformants were verified by sequencing. The *gfp-yabA* constructs were then integrated into the *amyE* locus of the *B. subtilis* ΔyabA strain (JJS142) by transformation, using spectinomycin (60 μgml^-1^) for selection. The presence of the integrated *gfp* fusions was verified by PCR amplification from the *amyE* flanking region and by the inability of the strain to produce amylase. For induction of the GFP-tagged YabA proteins, cells from overnight cultures grown at 37°C in LB supplemented with spectinomycin were diluted to OD600 = 0.01 in the same medium supplemented with xylose to 0.5% and grown until the OD600 reached 0.3–0.5. Cells were rinsed in MM and mounted on 1.2% pads. When required, cells were stained with FM4-64 (Molecular Probes) to visualize the cell membrane or with DAPI to visualize the nucleoid. Fluorescence microscopy was performed using Leica DMRA2. System control and image processing were performed using MetaMorph software.

### Microscopy Analysis of Sporulation

Cultures growing in a CH medium were induced to sporulate by transfer to resuspension medium (SM), essentially as described by Sterlini and Mandelstam (1969). Samples were taken at time T2 and mounted on 1.2% agarose pads. The cells were stained with DAPI to visualize the nucleoid, and with FM4-64 (Molecular Probes) to visualize the cell membrane. Fluorescence microscopy was performed on a Leica DMR2A using a 100 UplanAPO objective with an aperture of 1.35 and equipped with CoolSnap HQ camera (Roper Scientific). System control and image processing were performed using MetaMorph software. The indicated counts of cells were obtained from three to four independent experiments performed on over 400 cells per strain.

### Sporulation Efficiency

To quantify spore formation, cells were induced to sporulate by nutrient exhaustion in Difco sporulation medium (DSM) (Schaeffer et al., 1965). Overnight precultures were used to inoculate liquid DSM and incubated at 37°C until OD600 stop to increase. From this point (taken as T0) samples were grown for 18 h. Serial dilutions were plated on LB before and after a 20 min heat shock at 80°C. Colonies were counted after 24 h of incubation at 37°C, and the percentage of spores was calculated as the ratio of colonies forming units in heated and unheated samples.

### Luciferase and GFP Transcriptional Fusions

To analyze genes expression, *spoIIA* and *spoIIID* transcriptional fusions with the butterfly luciferase gene *luc* (Bidnenko et al., 2017) were used to transform *B. subtilis* strains where they integrated by single crossover. This event reconstructs natural regulatory region of a gene upstream the fusion and an intact copy of gene downstream. For the detection of luciferase activity, strains were grown in LB medium until exponential phase (OD600 0.4–0.5). The cells were centrifuged and re-suspended in fresh DSM media to obtain OD600 1.0. The pre-cultures were next diluted in respective media to OD600 0.025. Then 200 μl were distributed into each well in a 96-well black plates (Corning) and 10 μl of luciferin were added to reach a final concentration of 1.5 mg ml^-1^ (4.7 mM). The cultures were incubated at 37°C with agitation in a Perkin Elmer EnVision 2104 Multilabel Reader equipped with an enhanced-sensitivity photomultiplier for luminometry. The temperature of the clear plastic lid was maintained at 37°C to avoid condensation. Relative Luminescence Units (RLUs) and OD600 were measured at 5-min intervals.

For *spo0A* expression, pBSBII-spo0A plasmid containing a GFP transcriptional fusion was used to transform *B. subtilis* strains. The plasmid was integrated by single crossover at chromosomal loci of the targeted gene. GFP expression was measured after induction of sporulation (T0) by the resuspension method using a TECAN instrument.

### Biofilm Formation

Strains were grown in LB to OD600 of 1.0. For biofilm formation, 10 μL of culture were added to 2 ml of MSgg medium [5 mM potassium phosphate (pH 7), 100 mM MOPS (pH 7), 2 mM MgCl2, 700 μM CaCl2, 50 μM MnCl2, 50 μM FeCl3, 1 μM ZnCl2, 2 μM thiamine, 0.5% glycerol, 0.5% glutamate, 50 μg mL^-1^ tryptophan, 50 μg mL^-1^ phenylalanine] (Branda et al., 2001) in glass tubes. The tubes were incubated without agitation at 30°C for 44 h. For quantification, the MSgg medium was eliminated and the biofilm was re-suspended in 1 ml of H_2_O to measure the optical density by spectrometry at 600 nm.

### Analysis of Air-to-Liquid Biofilm Pellicles

Strains were grown in LB to OD600 of 1.0 and inoculated in 12-well culture plates containing 3.5 ml of MSgg media at OD600 = 0.1. Cultures were maintained in a growth chamber at 28°C and 70% humidity for 48 h. Pellicles were brought up to the top of the wells by slowly adding MSgg media and peeled of onto a 2.5 cm diameter circular cover slide. The cover slides with intact biofilm pellicles were mounted onto an Attofluor Cell Chamber and stained with the FilmTracer FM 1-43 Green Biofilm dye (Thermo Fisher Scientific). Stained biofilms were observed using a spinning disk confocal microscope [Nikon Eclipse Ti-E coupled with CREST X-LightTM confocal imager; objectives Nikon CFI Plan Fluor 10X, DIC, 10x/0.3 NA (WD = 16 mm); excitation was performed at 470 nm and emission recorded at 505 nm]. Images were processed using IMARIS software (Bitplane, South Windsor, CT, United States). Biofilm images were quantified using the surface function in IMARIS (XTension biofilm). Biovolumes were calculated based on total volume (μm3) per area (μm2) from *n* ≥ 3 samples.

### Complex Colony Formation

Cells were grown in LB shaking at 37°C to OD600 of 1.0. 10 μL cultures were then spotted on a dried MSgg plate (1.5% agar) and incubated for 96 h at 30°C. Colonies were measured and photographed using a camera. For each sample, a representative image from 3 examined colonies is presented.

### YabT Overexpression

For YabT overproduction, *yabT* gene was amplified using oligonucleotides YabT-F-SalI and YabT-R-SphI (Supplementary Table S2); digested by SalI and SphI enzymes and cloned at pDG148 and pDG148F plasmids (Supplementary Table S3). Expression of the cloned *yabT* gene in *B. subtilis* was induced by 0.5 and 1 mM IPTG during sporulation and biofilm conditions. The expression of YabT was determined by inmunodetection. The FLAG-tagged YabT was visualized using the primary mouse ANTI-FLAG M2 monoclonal antibodies (Sigma-Aldrich; dilution 1:10,000) and the secondary goat peroxidase-coupled anti-mouse IgG antibodies (Sigma-Aldrich; dilution 1:20,000). The immuno-detection was developed using the kit Clarity Western ECL substrate (Bio-Rad) as per the manufacturer on a ChemiDoc (Bio-Rad) imaging system. The images were analyzed using Image Lab.

## Results and Discussion

### YabA Is Phosphorylated by YabT at Threonine 71 Within the Flexible Inter-Domain Region

**FIGURE 1:**
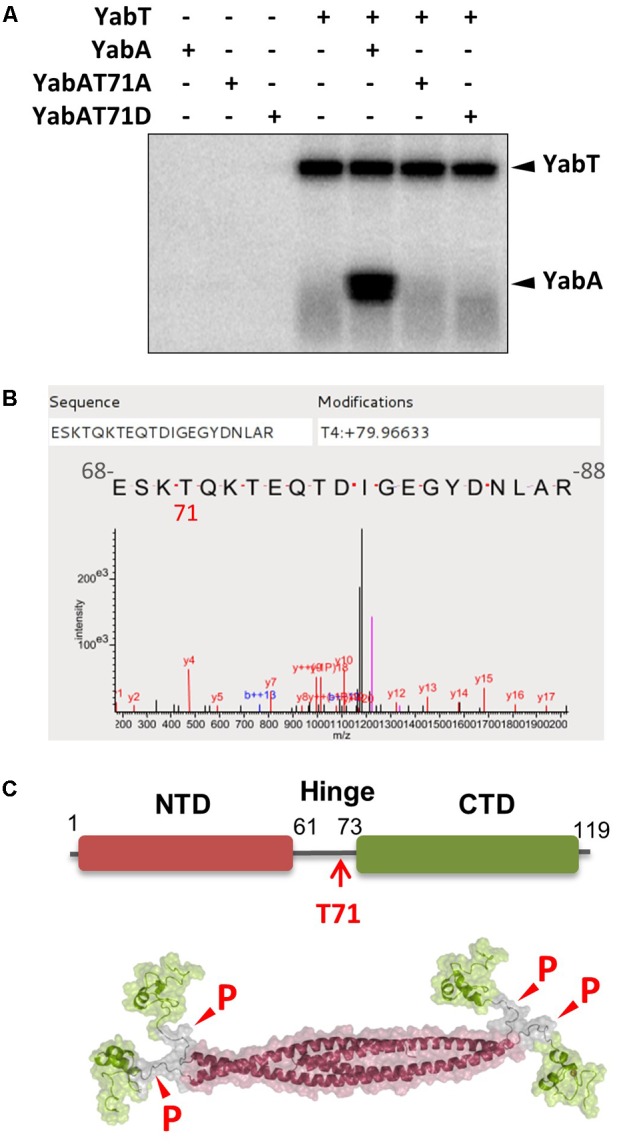
YabA is phosphorylated by the Ser/Thr kinase YabT at the residue T71 localized within the flexible inter-domain region. **(A)** *In vitro* phosphorylation assay of YabA, YabA-T71A (non-phosphorylatable) and YabA-T71D (phosphomimetic) in the presence of YabT. Presence of purified proteins is indicated with +/– above each line. Note that YabA often migrates as a doublet on SDS-PAGE gels that could correspond to isoforms of the protein (see also Supplementary Figure S1A). **(B)** Mass spectrometry detection of the phosphorylation site on YabA. *In vitro* phosphorylation reaction of YabA was carried out in the presence of YabT as described in materials and methods, only with non-radioactive ATP. Characterization of the phosphorylation site was performed by LTQ-Orbitrap and Q exactive and identification was realized by XTandemPipeline validated by MaxQuand (Supplementary Figure S2). The result shows an 80 Da molecular weight increment (corresponding to phosphate) at the position Threonine 4 of the peptide that corresponds with the Threonine 71 of the protein. **(C)** Mapping of the YabA T71 phosphosite within the flexible hinge region and YabA tetramer model showing the possible sites susceptible to phosphorylation.

A purified His6-tagged recombinant form of YabT was incubated with the native YabA protein at a 1:25 ratio in the presence of 32P-γ-ATP (**Figure 1**). YabT was found to auto-phosphorylate and to phosphorylate YabA *in vitro* (**Figure 1A**). The YabA phosphorylation site was identified by mass spectrometry as the threonine residue 71 (**Figure 1B**) and validated by MaxQuant (Supplementary Figure S2). To confirm the functional relevance of this phosphorylation site, this residue was replaced with the non-phosphorylatable residue alanine or the phospho-mimetic residue aspartate by site directed mutagenesis. The YabA-T71A and T71D mutant proteins were purified and tested *in vitro* for YabT-mediated phosphorylation. In both cases, the substitution of T71 prevented the transfer of a phosphoryl group by YabT, further indicating that T71 is the targeted phosphosite on YabA. YabA is composed of a core of four N-terminal α-helices, individually connected to a globular C-terminal region by a 14 residues long linking region. The mapping of T71 onto the YabA tetramer 3D-structure revealed that it is located within the flexible hinge region (**Figure 1C**). This extended linker was postulated to confer the high degree of flexibility observed in the YabA structure. These observations suggest that the phosphorylation at T71 could have an effect on the overall intrinsic flexibility of YabA.

### Phosphorylation at T71 Does Not Affect YabA Interaction Pattern

The flexibility of proteins was described to be important for the formation of protein complexes (Marsh and Teichmann, 2014). Previous work established that YabA interacts with multiple partners (Noirot-Gros et al., 2002, 2006; Felicori et al., 2016). The mapping of the binding surfaces on YabA highlighted two interacting domains. The free C-terminal domains of the YabA tetramer were found to be engaged in binding, in an exclusive manner, the initiator DnaA and the β-subunit of the replicative DNA polymerase DnaN, through a partially overlapping interacting surface. Similarly, an intersecting binding interface with the signaling and metabolic proteins TlpA and AcuB, respectively, was delineated on the tetrameric helical bundle formed by the N-terminal domain (Noirot-Gros et al., 2006; Felicori et al., 2016). We first investigated whether the phosphorylation of the residue T71 in the inter-domain hinge region of YabA could affect its protein-interaction landscape. The T71A and T71D substitutions were transferred on the *yabA* gene fused to the activating domain (AD) and binding domain (BD) of the yeast transcriptional factor GAL4 and the YabA-fusions were tested for their ability to interact with the different protein partners in a yeast two-hybrid assay (Supplementary Figure S3). The interaction patterns of the non-phosphorylatable and phosphomimetic forms of YabA were found to be similar to that of the wild type, indicating that the residue T71 was not likely to participate in modulating protein interactions.

### Phosphorylation at T71 Does Not Affect Replication Initiation Control

**FIGURE 2:**
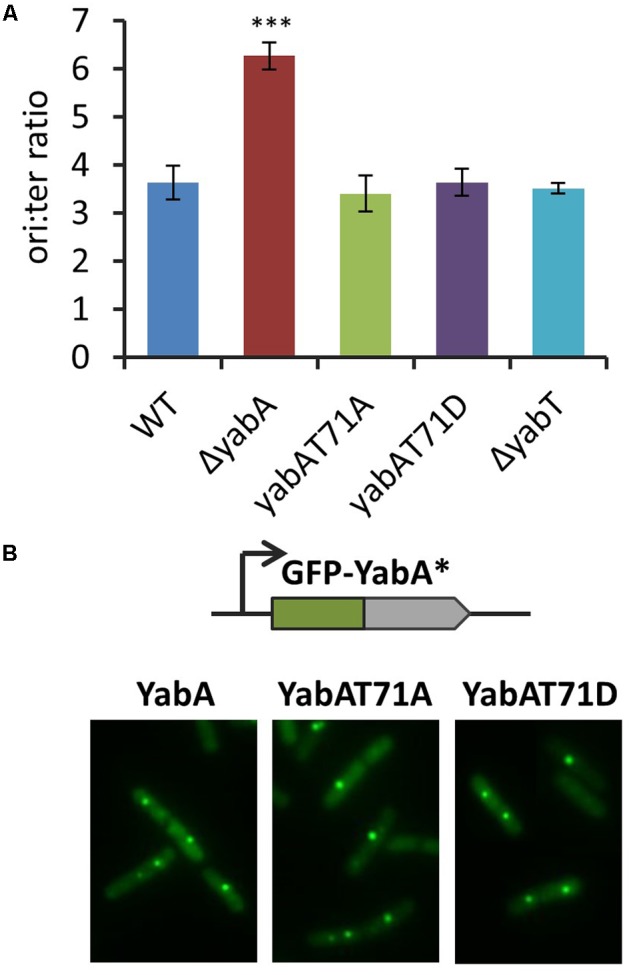
Phosphorylation of the YabA T71 residue is not involved in the replication initiation control. **(A)** Ori/ter values determined by Q-PCR on total DNA extraction of unsynchronized cells grown on LB media at 37°C up to OD ∼ 0.3. The asterisks indicate the result is statistically different compare to the control (*P* < 0.001 where *n* ≥ 3). The error bars represent standard error of the mean from 3 biological replicates. **(B)** YabA and YabA phospho-mutants localization. Fluorescence signals from GFP-YabA^∗^ (green) in living cells expressing YabA, YabA-T71A, or YabA-T71D proteins.

We then investigated the role of YabA phosphorylation in replication initiation control. *B. subtilis* mutant strains carrying mutations at the *yabA* genomic locus leading to T71A and T71D substitutions were constructed, and tested for potential initiation-defect phenotypes. Using origin-proximal (ori) and terminus-proximal (ter) primer pairs (Supplementary Table S2), we monitored the ratio of origin-to-terminus (ori-to-ter) genomic sequences as a means of detecting over-initiation events. In the Δ*yabA* null strain, the ori-to-ter ratio was twice as high as compared to the wild-type (**Figure 2A**), as already shown in previous studies (Soufo et al., 2008; Murray and Koh, 2014). However, the deletion of *yabT* as well as the expression of the non-phosphorylatable or the phosphomimetic versions of *yabA* gave rise to ori-to ter ratio similar to the wild type (**Figure 2A**). This indicated that the T71 residue was not involved in down-regulation of replication initiation and that YabT-dependent phosphorylation of YabA was not required for DnaA-mediated replication initiation control.

Previous studies established that YabA also exerts its function as part of a heterocomplex with DnaA and DnaN that localizes at the replication machinery. GFP-YabA fusion was found to form a discrete focus co-localizing with DnaN, and centrally localized at the nucleoid during most of the bacterial cell cycle (Noirot-Gros et al., 2002, 2006; Hayashi et al., 2005; Soufo et al., 2008). We constructed *B. subtilis* strains conditionally expressing a *gfp-yabA*, a *gfp-yabA*-T71A, or a *gfp-yabA*-T71D gene fusion from the ectopic chromosomal site *amyE*. These constructs were placed in their cognate wt-*yabA*, *yabA*-T71A, and *yabA*-T71D genetic backgrounds (Supplementary Table S1). Examination of the foci localization patterns revealed that the YabA subcellular localization remained unaffected by the substitutions at T71 (**Figure 2B**). Altogether, these experiments showed that YabA-T71A and YabA-T71D have retained all the functional properties of the wild type YabA, suggesting that phosphorylation of T71 is not involved in initiation control. Together with the retention of ability to interact with all there protein partners, these results provided evidence that the substitutions at T71 did not affect the structural and functional integrity of YabA.

### YabA Phosphorylation Enhances Sporulation

**FIGURE 3:**
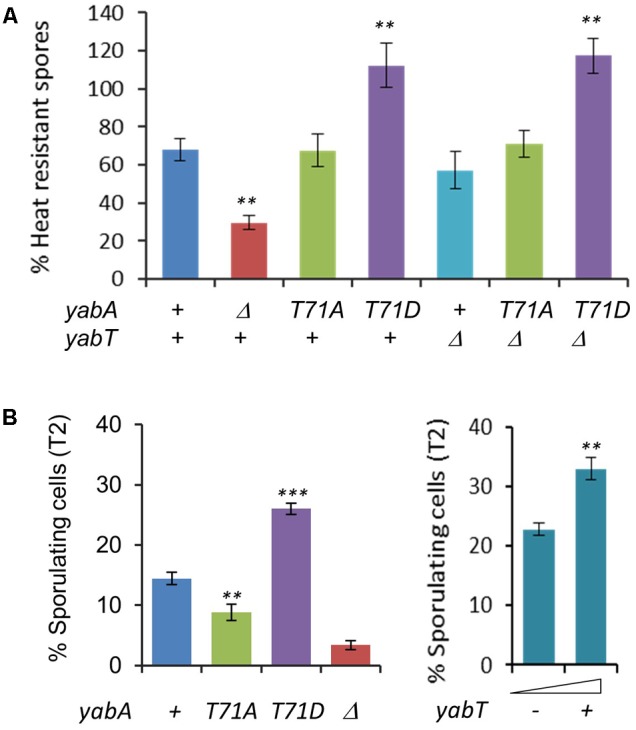
YabA phosphorylation enhances sporulation. **(A)** Quantification of heat resistant spores of wild type *Bacillus subtilis* and strains Δ*yabA*, *yabA*-T71A, *yabA*-T71D, Δ*yabT* and double mutants (Δ*yabT*-*yabA*-T71A and Δ*yabT*-*yabA*-T71AD) in Difco sporulation media DSM at T18. Spores counts are expressed as % of total number of viable cells. Error bars represent standard error of the mean from 3 biological replicates. *P*-values are indicated by asterisks (^∗^*P* < 0.05; ^∗∗^*P* < 0.01, and ^∗∗∗^*P* < 0.001). **(B)** Percentages of sporulating cells observed by microscopy at stage T2 after induction of sporulation by the resuspension method. Error bars represent standard error of the mean from 5 biological replicates. Over 400 cells were counted for each strain and replicate **(Left)**. Effect of YabT overexpression using a pDG148-flag-yabT plasmid inducible by IPTG. Sporulation was carried out by the resupension method as previously and expression of YabT was induced by adding 1 mM of IPTG at T0 (resuspension). Percentage of sporulating cells was calculated at T2 **(Right)**. Error bars represent standard error of the mean from 3 biological replicates. *P*-values are indicated by asterisks (^∗^*P* < 0.05, ^∗∗^*P* < 0.01, and ^∗∗∗^*P* < 0.001).

Analysis of the transcriptional profiles of *yabT* from transcriptomes of *B. subtilis* cells exposed to many environmental and nutritional conditions revealed that *yabT* was expressed at an early stage of sporulation (data available at^6^). We examined the physiological conditions under which the *yabT* and *yabA* genes exhibited correlated expression profiles (Supplementary Figure S4). While *yabA* is highly expressed during all conditions, the expression of *yabT* is triggered only (i) during sporulation, with a maximum expression peak 3 h after the onset of sporulation, (ii) during biofilm formation, and (iii) after glucose exhaustion (Supplementary Figure S4). Interestingly, YabT has already been shown to be involved in the chromosome integrity control during sporulation. We therefore investigated whether YabA phosphorylation could play a role during sporulation. Our wild type, non-phosphorylatable and phosphomimetic *yabA* strains were induced to sporulate by the exhaustion method in DSM media. The point when cells stop to divide is considered as the onset of sporulation (T0). We determined the survival efficiency of mature spores, expressed as the ratio of heat-resistant spores obtained 18 h after T0 (**Figure 3A**). The *yabA* deleted strain was found to be significantly affected in sporulation, reaching 30% of efficiency compared to 70% of resistant spores for the wild type (*P* = 0.0038), indicating that *yabA* could play a role during sporogenesis. We observed that the *yabA*-T71A strain exhibited the same level of sporulation as the wild-type strain (∼70%) while the *yabA*-T71D mutant reached the highest sporulation efficiency slightly above 100% (*P* = 0.0043), indicating that YabA phosphorylation at T71 could enhance sporulation. Note that the sporulation efficiencies greater than 100% found in the *yabA*-T71D strain derivatives might result from the existence of short chains or doublets leading to an artificial excess of spore counts after heating treatment. The Δ*yabT* mutant strain produced a final yield of mature spores similar to the wild-type, as previously observed. Examination of the Δ*yabT yabA*-T71D double mutant revealed that the phosphomimetic mutant derivative of *yabA* exhibited the same propensity to stimulate sporulation in a *yabT*-deficient genetic background (Bidnenko et al., 2013). This indicates that *yabT* and *yabA* are part of the same pathway and suggests that YabT-dependent phosphorylation of YabA has an effect on sporulation (as further demonstrated below).

The effect of YabA phosphorylation in spore yields was visible in the early stage of the sporulation process by microscopy analysis (Supplementary Figure S5A). At T2, about twice as many cells were committed to sporulation (i.e., exhibited an asymmetrical septum) in the *yabA*-T71D (27%, *P* < 0.0001) as compared to the wild-type (14.5%). In contrast, in the *yabA*-T71A strain the number of stage T2-sporulating cells was reduced by 1.6-fold (9%, *P* = 0.006) compared to the wild type (**Figure 3B** and Supplementary Figure S5A). The Δ*yabA* strain showed the most severe defect in spore production with a 4.25-fold reduction (3.4%) compared to the wild type (*P* < 0.0001). The sporulation increase phenotype observed in *yabA*-T71D was maintained over 6 h up to the production of mature spores (Supplementary Figure S5C). Altogether, the results obtained at early (T2) and late (T8) sporulation stages suggest that the phosphorylation of YabA at T71 enhances the commitment of *Bacillus* cells to sporulation, leading to an increase in spore production, while the absence of phosphorylation only delays the entry into sporulation without affecting the final spore yield.

Our next question was to determine whether this phosphorylation-mediated stimulation of sporulation is related to YabT activity. The *yabT* gene was inserted in a plasmid under the transcriptional control of the IPTG-inducible P*spac* promoter. The resulting construct pDG148flag-yabT, which allows the expression of the Flag-tagged YabT was then introduced in the wild-type *yabA* strain. Increased intracellular levels of YabT at T2 in crude extracts of cells harboring the pDG148-flag-yabT construct was confirmed by a Western blotting assay (Supplementary Figure S5D). Samples induced by the addition of IPTG 0.5 or 1 mM yielded signals of comparable intensities, indicating that YabT was efficiently produced *in vivo* and was not degraded by host proteases. Similar to *yabA*-T71D, we observed that the number of stage T2-sporulating cells was enhanced about 60% upon addition of IPTG (*P* = 0.0075), indicating a correlation between the level of YabT expression and the efficiency of entry into the sporulation process (**Figure 3B** and Supplementary Figure S5B). A comparable increase in sporulating cells at T2 was observed with statistical significance (*P* = 0.04) upon the expression of the untagged YabT protein in *yabA* wild type cells, that was abolished in YabA-phosphorylation mutant cells, suggesting that the increase requires the integrity of the YabA phosphosite and did not result from unrelated YabT function (Supplementary Figure S5B). Taken together, our findings highlighted a correlation between increased intracellular level of YabT, YabA phosphorylation and efficiency of entry into the sporulation process that support the hypothesis that the YabT-mediated phosphorylation of YabA at T71 plays a regulatory role during sporulation.

### Effect of YabA Phosphorylation at T71 on Sporulation Early Developmental Program

**FIGURE 4:**
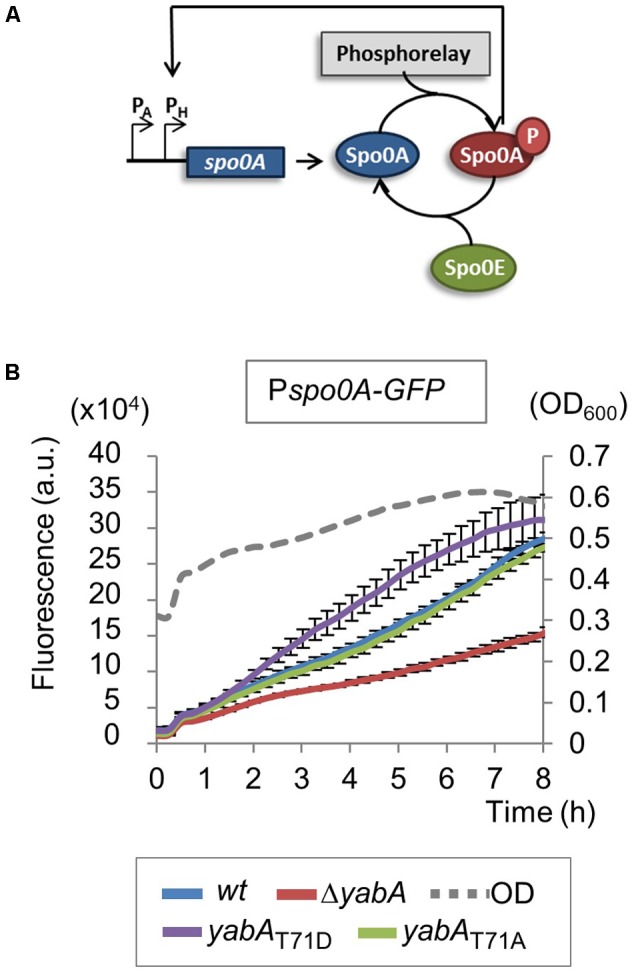
Expression of *spo0A* increases in *yabA*-T71D strain. **(A)** Schematic representation of *spo0A* regulatory loop. When phosphorylated, Spo0A is accumulated and activates its own expression. **(B)** Expression of *spo0A* was measured after induction of sporulation by the resuspension method using a P*spo0A-gfp* transcriptional fusion. *Bacillus* strains expressing the *yabA* wild type or mutated derivatives are indicated by colors. Error bars represent standard error of the mean from 3 biological replicates.

**FIGURE 5:**
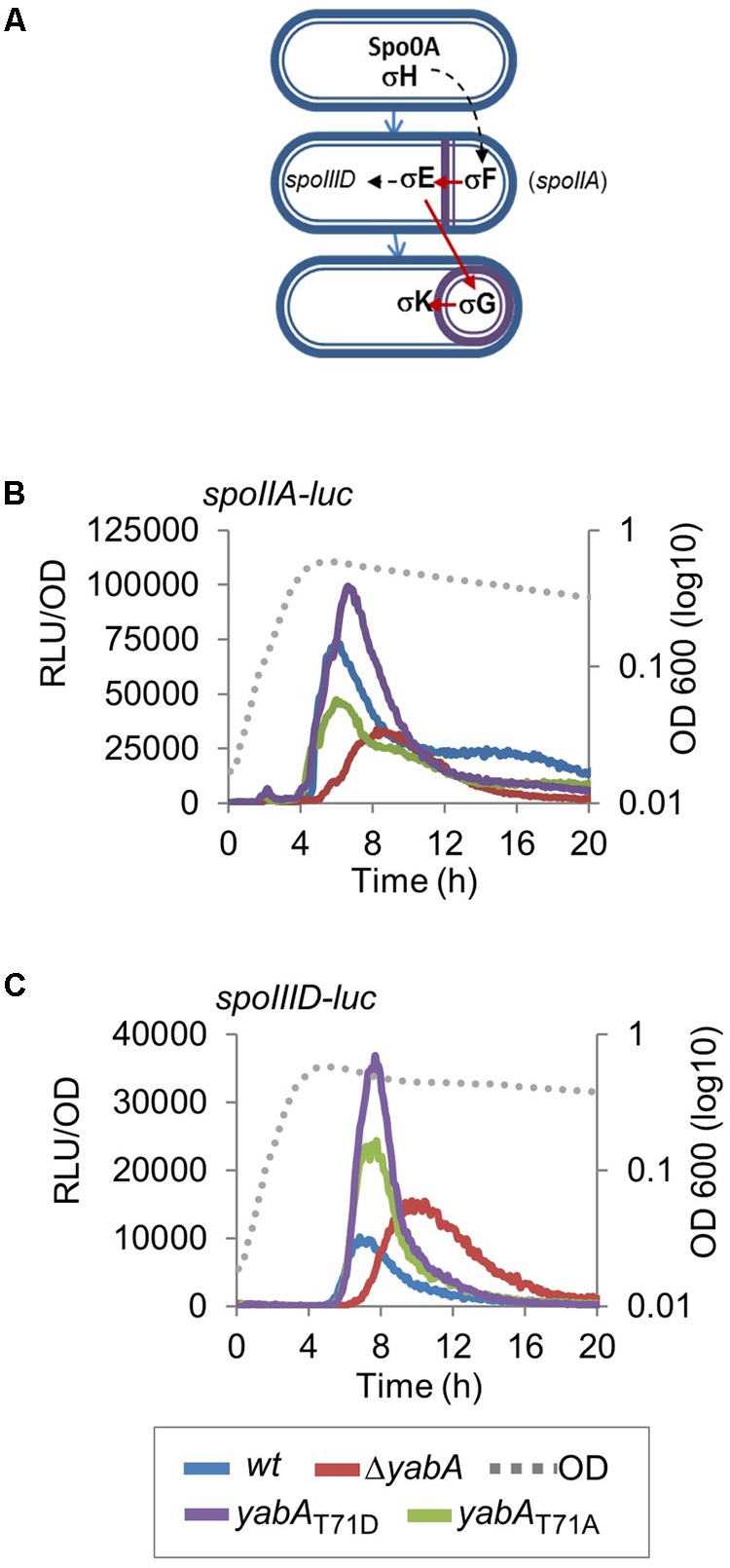
Effect of YabA phosphorylation at T71 on sporulation early developmental program. **(A)** Illustration of sigma factors cascade controlling the sporulation process. **(B)** Luciferase was placed under the control of P*spoIIA* promoter, which drives the expression of *spoIIA*, an early-stage sporulation gene whose expression is directly regulated by σH, and **(C)** P*spoIIID* promoter, which drives the expression of *spoIIID*, a middle-stage sporulation gene whose expression is directly regulated by σE. *Bacillus* strains expressing the *yabA* wild type or mutated derivatives are indicated by colors.

Sporulation is an adaptive developmental program engaged when the bacteria encounter conditions of starvation, leading to the liberation of highly resistant spores (Piggot and Hilbert, 2004). Sporulation in *B. subtilis* is a strictly regulated process linked to the phosphorylation of the master regulator Spo0A, modulated by a multicomponent phosphorelay system (Sonenshein, 2000; Stephenson and Lewis, 2005; Vishnoi et al., 2013). Only cells expressing a high level of Spo0A∼P can initiate sporulation (Fujita and Losick, 2005; Worner et al., 2006; Chastanet and Losick, 2011). Then, compartment-specific transcriptional programs that drive sporulation are activated by successive sporulation-specific sigma factors σF, σE, σG, and σK (Steil et al., 2005). We evidenced that the phosphorylation state of YabA differentially affects sporulation at an early stage. To better understand the role of YabA phosphorylation at the onset of sporulation, we constructed a P*spo0A-gfp* transcriptional fusion and compared the expression profiles in our *yabA*-T71 mutant strains after initiation of sporulation (**Figure 4**). When cells enter the sporulation developmental program, phosphorylated Spo0A accumulates and activates its own expression (Molle et al., 2003; Fujita and Losick, 2005; **Figure 4A**). Indeed, the fluorescence profile of the gfp expressed from Pspo0A in the wild-type yabA background increased with time (**Figure 4B**). Expression from Pspo0A in the yabA-T71A strain was found to be similar to that obtained in the wild-type background, while it is reduced twofold in the absence of yabA, hinting at a regulatory role of yabA in spo0A transcription. Interestingly, expression of the spo0A promoter was most efficient in the presence of the YabA-T71D protein, suggesting that indeed, accumulation of Spo0A∼P was enhanced in this strain. To further investigate the role of YabA phosphorylation on the sporulation transcription program, we examined the expression of promoters driven by the early sporulation specific sigma factors σF and σE. At the onset of sporulation, elevated levels of Spo0A-P activates the transcription factor σH that will trigger the expression of numerous genes including the spoIIA operon, encoding for σF as well as its regulators, and σE (Piggot and Hilbert, 2004). Both sporulation σ factors are first held in an inactive form in the mother cell and their activation is linked to the formation of the asymmetric division septum. Activation of the σF factor occurs in the forespore and is followed by the activation of σE through cleavage of the pro-σE form (**Figure 5A**; Hilbert and Piggot, 2004; Piggot and Hilbert, 2004). To investigate the impact of YabA phosphorylation on the early steps of sporulation, we constructed luminescent reporters for the promoter PspoIIA, controlled by the Spo0A-P-regulated σH factor, as well as for the promoter PspoIIID, controlled in a σE-dependent manner. Both genes were transcriptionally fused with the *luc* gene encoding the firefly luciferase. Luciferase expression profiles were examined for 20 h after sporulation. In the wild type and yabA-T71A and -T71D genetic backgrounds the expression peak of the PspoIIA-luc and PspoIIID-luc fusions occurred at a similar time but not with a similar intensity (**Figures 5B,C**). Compared to the wild-type, the expression of PspoIIA-luc was diminished in the yabA-T71A strain but was significantly higher in a yabA-T71D background, consistent with sporulation kinetics and spore yields obtained earlier (**Figure 3**). Indeed, the lower expression in yabA-T71A could account for the sporulation delay observed in this strain (**Figure 3B**), while the elevated expression in yabA-T71D is in accordance with the increased sporulation efficiency triggered by the phosphomimetic mutation. PspoIIID-luc expression is the highest in the yabA-T71D background. These observations are in agreement with the hyper-sporulation phenotype exhibited in the yabA-T71D strain. We also noticed that the expression of these early promoters was strongly delayed in the absence of YabA, which is consistent with a low sporulation efficiency of the ΔyabA mutant. Taken together, these results highlighted a regulatory role of YabA phosphorylation at residue T71 during the early sporulation program. The stimulation of the expression of the early sporulation spoIIA gene indicates a positive activation of this σH-dependent promoter. These results provide additional evidence for enhanced levels of Spo0A-P mediated by YabA phosphorylation.

### YabA Phosphorylation Inhibits Biofilm Formation

**FIGURE 6:**
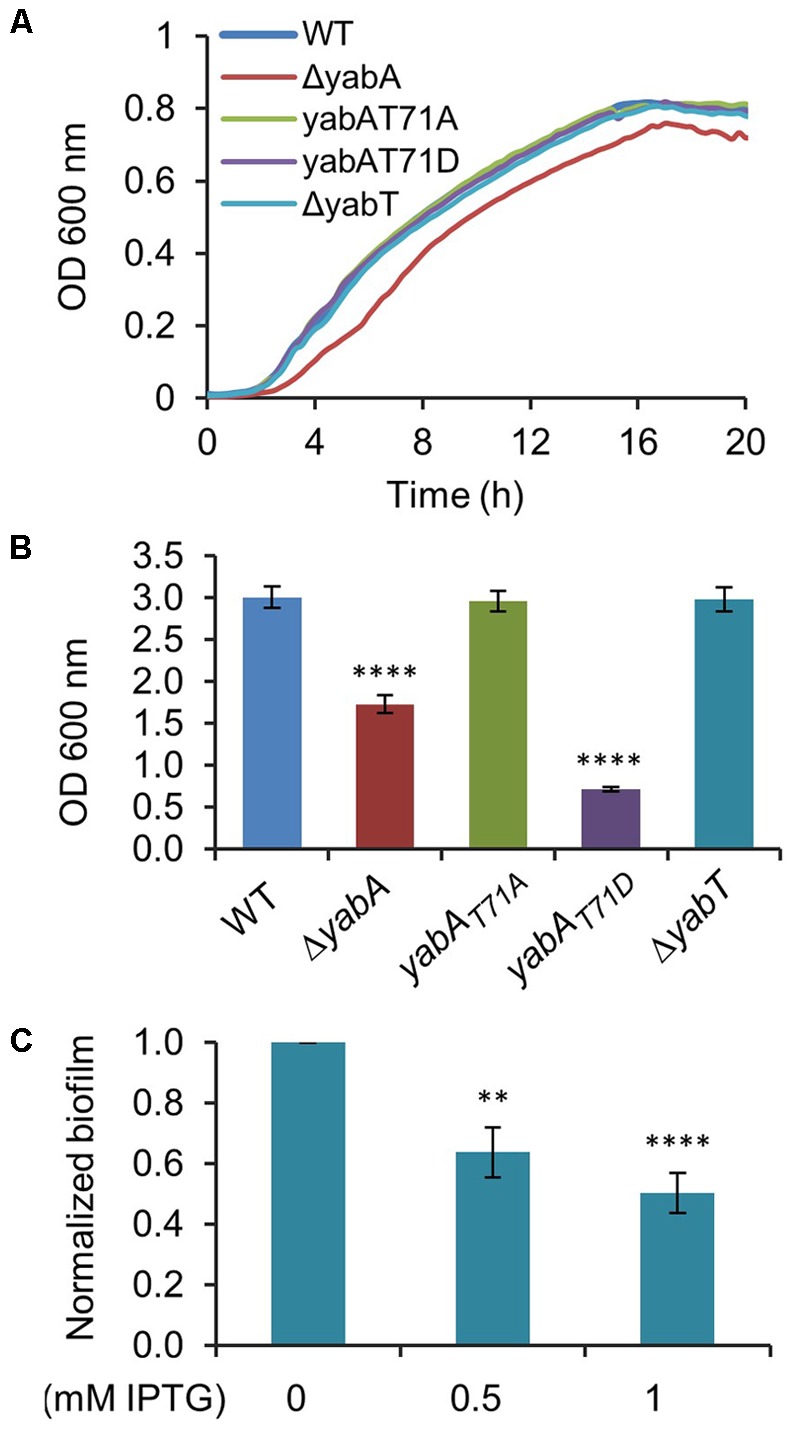
YabA phosphorylation induces a defect in biofilm formation. **(A)** Growth curves of *B. subtilis* strains in MSgg medium. **(B)** Measurement of optical density at 600 nm of re-suspended biofilm cells. Error bars represent 3 independent experiments with 4 biological replicates. **(C)** Effect of YabT overexpression using pDG148-flag-yabT plasmid inducible by IPTG in biofilm formation. Biofilms were carried out as previously, with the only difference of adding 0.5 mM or 1 mM of IPTG to MSgg media to induce *yabT* expression. Error bars represent of the standard error of the mean over 5 biological replicates. *P*-values are indicated by asterisks (^∗∗^*P* < 0.01 and ^∗∗∗∗^*P* < 0.001).

**FIGURE 7:**
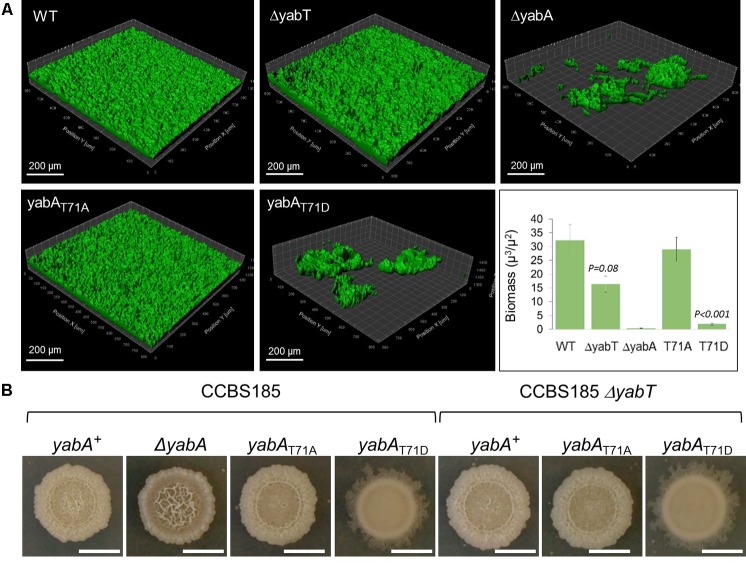
Structure and morphology of air-liquid biofilms. **(A)** 3D-volumes of the wild-type, Δ*yabA*, *yabA*-T71A, *yabA*-T71D, Δ*yabT* and double mutants (Δ*yabT*-yabA-T71A, and Δ*yabT*-*yabA*-T71AD) derivatives of CCBS185 strain. 3D-volumes were imaged and analyzed using the surface function of IMARIS. Biovolumes based on total volume (μm3) per area (μm2) are plotted. Error bars represent of the standard error of the mean (*n* ≥ 3). **(B)** Colony morphology of wild-type *B. subtilis*, and strains Δ*yabA*, *yabA*-T71A, *yabA*-T71D, Δ*yabT*, and double mutants (Δ*yabT*-*yabA*-T71A and Δ*yabT*-*yabA*-T71AD). Representative images of colonies incubated in MSgg plates for 4 days at 30°C.

Sporulation is an energy consuming process which becomes irreversible after engulfment of the prespore by the mother cell. Early after starvation, cells might also adopt other solutions to survive, such as cannibalism, competence or biofilm formation (Chai et al., 2008; Vlamakis et al., 2013). The decision process is linked to the Spo0A∼P to Spo0A ratio, which gradually increases after starvation. Biofilm formation and sporulation are repressed when Spo0A∼P is low (Hamon and Lazazzera, 2001; Chai et al., 2008). At intermediate levels of Spo0A∼P, the biofilm developmental program is induced, whereas only cells expressing high levels of Spo0A∼P can enter into sporulation (Chai et al., 2008). We hypothesized that the phosphorylation of YabA we earlier found to correlate with enhancement of sporulation and Spo0A-mediated gene expression would, as a corollary, affect biofilm formation. *B. subtilis* is described to develop different biofilms from surface-associated to air-liquid pellicle (Vlamakis et al., 2013). We looked at the capacity of our strains to form air-liquid biofilm. We first verified that all strains were able to grow in MSgg medium: only the growth of the Δ*yabA* strain was slowed down (**Figure 6A**). When cultured in glass tubes, the *yabA*-T71A and Δ*yabT* mutant derivatives strains exhibited similar re-suspended biofilm cell densities than the wild type *yabA*, while biofilms formed by the Δ*yabA* and the *yabA*-T71D strains were 44 and 77% less, respectively, suggesting a negative role of YabA phosphorylation in biofilm formation (**Figure 6B**). Biofilm cell density was also investigated upon overexpression of a Flag-tagged YabT. We found that overexpression YabT correlated with a decrease of biofilm cell density (**Figure 6C**), supporting that YabT-dependent phosphorylation of YabA may play a role in tuning of biofilm formation. The tridimensional structures of air-liquid biofilms pellicles formed in 12-well culture plates were visualized by confocal microscopy and their biovolume quantified using the software Imaris (**Figure 7A**). Again, the wild type *yabA*, the *yabA*-T71A and Δ*yabT* displayed similar biofilm structures with statistically comparable biovolumes. Contrastingly, both the YabA-T71D and the Δ*yabA* strains totally failed to form a pellicle (**Figure 7A**).

We set out to confirm our findings from the angle of colony architecture formed on MSgg medium (**Figure 7B**). *B. subtilis* macrocolonies exhibit sophisticated three-dimensional structural designs on agar media, reflecting their ability to produce exopolysaccharides (EPS) polymers that compose the biofilm matrix, as well as other proteins important for the biofilm structure integrity (Branda et al., 2006; Romero et al., 2010). The enhanced matrix production in biofilms has been attributed to the derepression of the *epsA-O* and *tapA-sigW-tasA* operons, which are normally inhibited by the transcriptional regulator SinR (Kearns et al., 2005; Leiman et al., 2014). This is achieved via the protein SinI, an antagonist of SinR, which alleviates the repression of the genes encoding the components of the biofilm matrix and which expression is activated at a low-to-intermediate level of Spo0A∼P (Supplementary Figure S6; Kearns et al., 2005; Chai et al., 2008; Chu et al., 2008). We observed a clear difference in colony morphology in the absence of YabA compared to the wild-type strain. The Δ*yabA* strain displayed a hyper-wrinkled morphotype, indicative of a high production of extracellular matrix (ECM) associated with localized zones of cell death that trigger mechanical forces to form wrinkled structures (**Figure 7B**; Spiers et al., 2003; Spiers and Rainey, 2005; Asally et al., 2012; Ghosh et al., 2013; Leiman et al., 2014). Our observations suggested that the absence of YabA could lead to the de-regulation of the EPS synthesis pathway. However, we also observed that the overall production of biofilm cell mass is diminished in the Δ*yabA* (**Figure 6B**). This reduction of cell mass in the Δ*yabA* biofilms could be attributed to the decrease of cell growth observed in this strain (**Figure 6A**), associated with cell death during static growth in liquid as well as on semi-solid agar surfaces. Observation of the macrocolony morphology of the *yabA*-T71A revealed only a subtle difference compared to that of the wild-type strain, the later displaying a slight wrinkle phenotype at the center of the colony, not found in *yabA*-T71A (**Figure 7B**). Interestingly, the *yabA*-T71D strain exhibited a smooth phenotype indicative of a defect in EPS production (**Figure 7B**). The deletion of the *yabT* gene induced a similar absence of wrinkles as in *yabA*-T71A. This phenotype is not affected when combined with the non-phosphorylatable mutation of *yabA* (Δ*yabT yabA*-T71A strain), while the Δ*yabT yabA*-T71D strain exhibited the same smooth colony structure as the *yabA*-T71D strain. This indicated that YabT and YabA act in the same pathway regarding biofilm formation. Altogether, these results suggest that the YabA phosphorylation at T71 mediated by YabT prevents biofilm formation. Our results further support a role of YabT-mediated phosphorylation of YabA in negatively regulating biofilm formation through a Spo0A-directed repression of transcription of biofilm operons.

## Conclusion

Our results highlight the existence of a role of the phosphorylation of the replication initiation controller YabA in *B. subtilis* cell fate decision, through the modulation of Spo0A-P intracellular levels. YabA phosphorylation is carried out by the Ser/Thr kinase YabT, expressed at the early stage of sporulation and during biofilm formation. We established that YabA phosphorylation correlates with high cellular levels of Spo0A-P, leading to sporulation stimulation. A consequence of elevated levels of activated Spo0A is the prevention of the expression of the anti-repressor SinI, leading to full repression of the EPS operon by the master regulator SinR (Chai et al., 2008; DeLoughery et al., 2016). This could provide a foundation for the YabA-phosphorylation mediated inhibition of biofilm formation.

The mechanism underlying this novel role of YabA remains to be further characterized. However, the discovery that YabA can carry out another function that seems to be unrelated to its primary function in replication initiation control contributes to the complexity of its biological role in *B. subtilis*. Many proteins are found to be multifunctional in eukaryotic but also bacterial cells. These multitasking proteins exert a variety of activities in different biological pathways, thus contributing to cellular network complexity (Jeffery, 2016). Post-translational protein modifications are important mechanisms that regulate protein activities in a reversible way. The role of protein phosphorylation in triggering a switch from a given function to another unrelated function is documented, most particularly in eukaryotes (Gancedo et al., 2016; Jeffery, 2016). However, bacterial proteins were also described as performing more than one function spanning processes such as metabolism, virulence, stress or even DNA maintenance (Huberts and van der Klei, 2010; Mani et al., 2015).

YabA belongs to a family of replication controllers, largely conserved in low GC sporulating or non-sporulating Gram-positive bacteria. The YabT-targeted residue T71 of YabA is located in a structurally flexible domain linking the well conserved N-terminal and C-terminal parts. This flexible hinge is rather divergent among the YabA family of proteins and the residue T71 is not conserved in many other related bacteria. This novel function of YabA might provide *B. subtilis* cells with a useful cross talk between the different cellular processes for fine tuning cell fate decision and to better respond to environmental changes.

## Author Contributions

SP, TGG,MV, andMFNGcontributed to the experimental design. SP, TGG, MV, VB, AD, CH, SCS, and MFNG performed the biological experiments. SP, TGG, IM, and MFNG contributed to critical analysis of the data. SP, TGG, and MFNG wrote the manuscript. All authors have read and approved the final manuscript.

## Conflict of Interest Statement

The authors declare that the research was conducted in the absence of any commercial or financial relationships that could be construed as a potential conflict of interest.

## Footnotes

1 http://www.thegpm.org/TANDEM/

2 http://www.uniprot.org/

3 http://pappso.inra.fr/bioinfo/

4 http://pappso.inra.fr/bioinfo/xtandempipeline

5 http://www.maxquant.org

6 www.basysbio.eu/bsubtranscriptome/seb

## Abbreviations

ECM: extracellular matrix
EPS: exopolysaccharides
SDCM: spinning disk confocal microscopy.
