# Supplementary material for: Phosphorylation of the Bacillus subtilis Replication Controller YabA Plays a Role in Regulation of Sporulation and Biofilm Formation
Source: Front Microbiol. 2018 Mar 21;9:486. doi: 10.3389/fmicb.2018.00486 (PMC5871692; doi:10.3389/fmicb.2018.00486)
Supplement: Supplementary file 1 [file Data_Sheet_1.PDF]

Supplementary Material

Phosphorylation of the Bacillus subtilis replication controller YabA reveals its role in regulation of sporulation and biofilm formation First

Tránsito García García1, M. Ventroux1, A. Derouiche2, V. Bidnenko1, S.F. Correia Santos, C. Henry1, I. Mijakovic2, M-F Noirot-Gros3* and S. Poncet1*

1 Micalis Institute, INRA, AgroParisTech, Université Paris-Saclay, Jouy-en-Josas,France

2 Systems and Synthetic Biology, Chalmers University of Technology, Göteborg, Sweden

3 Argonne National Laboratory, Biosciences Division, Argonne, IL, United States of America

*** Correspondence:**

Corresponding Authors: Sandrine Poncet : sandrine.poncet-mouturat@inra.fr; Marie-Françoise Noirot-Gros : mnoirot@anl.govSupplementary Data

Keywords: Bacillus subtilis, Ser/Thr kinase, Sporulation, Biofilm, Replication initiation control.

1. Supplementary Figures

1.1. Supplementary Figure 1.

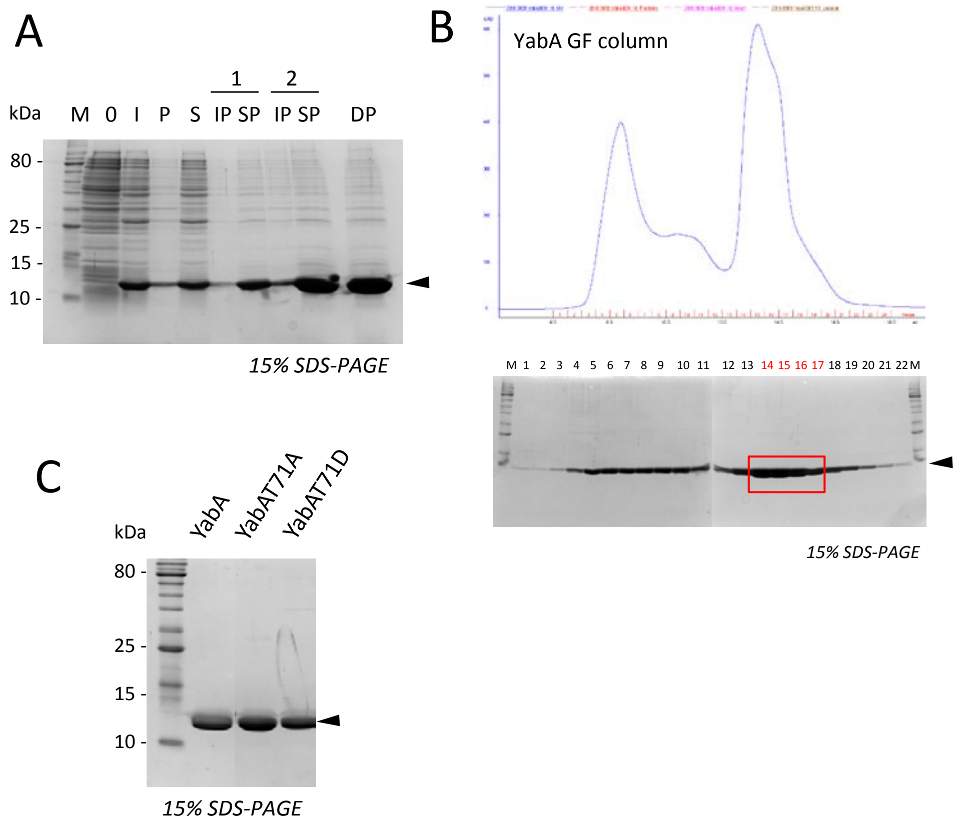

Figure S1. YabA protein purification (**A**) SDS-PAGE showing YabA purification by precipitation with ammonium sulfate 20 % (band at ~14 kDa). The soluble part containing YabA protein was separated by centrifugation and dialyzed in 50% glycerol. (**B**) Superdex 200 10/300 GL gel filtration of YabA *(top)* and SDS-PAGE of collected fractions; fractions 14 to 17 were pooled and dialyzed *(bottom)* (**C**) SDS-PAGE showing YabA, YabA-T71A and YabA-T71D proteins. *SDS gel legends figure A*: M = M.W. marker, 0 = before induction, I=after induction, S= supernatant, P= insoluble pellet, IP=insoluble part, SP= soluble part, DP= dialyzed protein. Note that YabA often migrates as a doublet on SDS-page gels. This could not be fixed by increasing the amount of reducing agent in the loading buffer indicating that it does not result from the oxidation state of the protein. However, YabA was found to be highly flexible (Felicoli et al., 2016), suggesting that the doublet could correspond to isoforms of the protein.

1.2. Supplementary Figure 2.

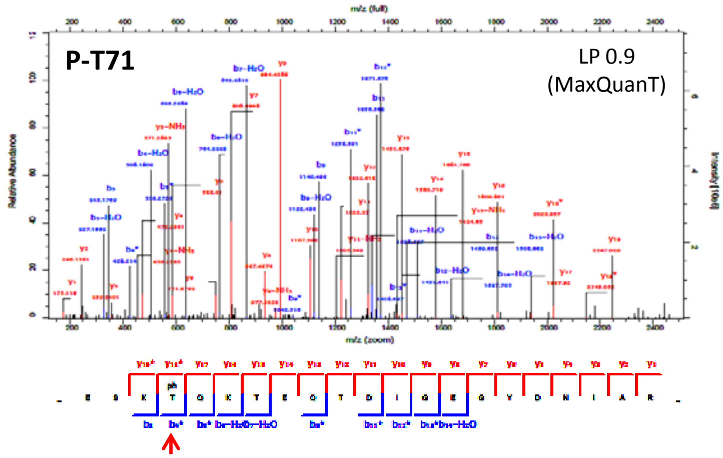

Figure S2. MaxQuant validation of YabA phosphorylation at T71: Spectra view from MaxQuant that confirms the position T71 with a very good score of localization probability (0.9, over a maximum of 1).

1.3. Supplementary Figure 3.

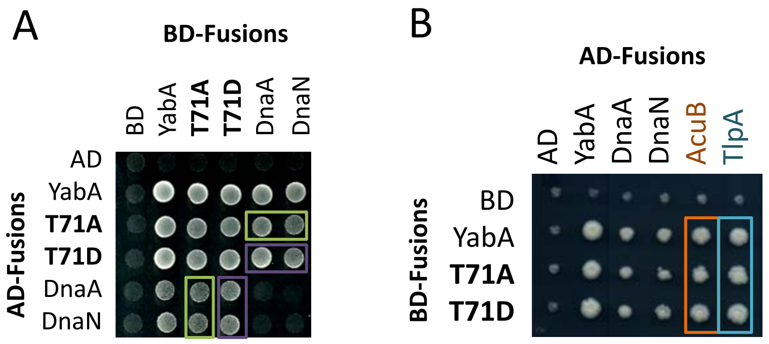

Figure S3. **YabA T71 mutants are not affected in their ability to interact with YabA partners**. (A) Interaction between YabA non-phosphorylatable (T71A) and phosphomimetic (T71D) derivatives with DnaA and DnaN detected by yeast-two-hybrid. All genes were expressed in fusion with both the binding domain (BD) and activating domain (AD) of GAL4 and tested for interaction in a yeast two-hybrid matrix assay. (B) YabA-T71A and YabA-T71D mutant baits were still proficient for interaction with the YabA partners AcuB and TlpA in a yeast two-hybrid assay.

1.4. Supplementary Figure 4.

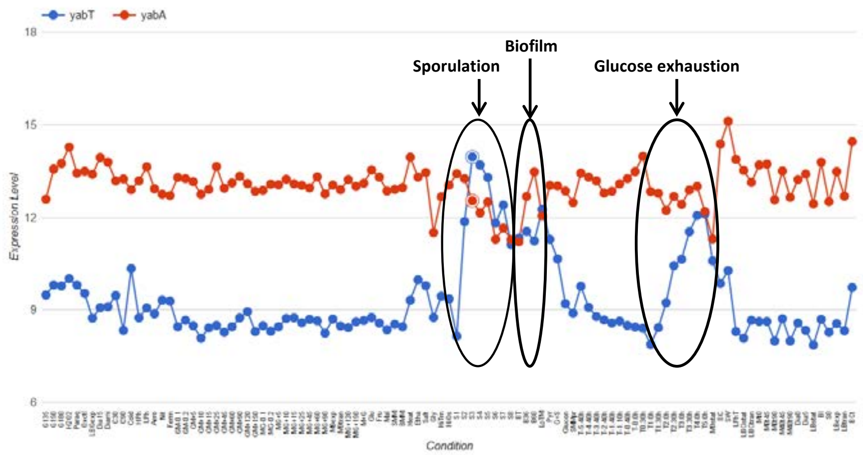

Figure S4. ***yabT* and *yabA* are co-expressed during sporulation, biofilm and glucose exhaustion.** Transcriptomic data over >100 conditions extracted from Nicolas et al. (BaSysBio). Graphic obtained from Subtiwiki. Expression of *yabA* is shown in red and *yabT* in blue.

1.5. Supplementary Figure 5.

Figure S5. YabT-mediated phosphorylation of YabA at 71 enhances sporulation. (A) A sample of sporulating cells observed at T2 with FM4-64 to stain membranes and DAPI to stain DNA. (B) Effect of YabT overexpression using a pDG148-yabT plasmid inducible by IPTG in WT, yabA-T71A and yabA-T71D backgrounds. (C) Kinetics of sporulation of wild-type *B. subtilis* and strains *yabA*-T71A and *yabA*-T71D in resuspension medium from stage T0 to T6. Sporulation was followed by microscopy and the percentage of sporulating cells was calculated for each time. (D) Expression of YabT was confirmed by western blot, using ANTI-FLAG M2 monoclonal antibodies (Sigma-Aldrich; dilution 1:10.000) as primary antibody and revealed by the secondary goat peroxidase-coupled anti-mouse IgG antibodies (Sigma-Aldrich; dilution 1:20.000), from equivalent amounts of total cells lysates of *B. subtilis* expressing YabT.

Supplementary Figure 5.

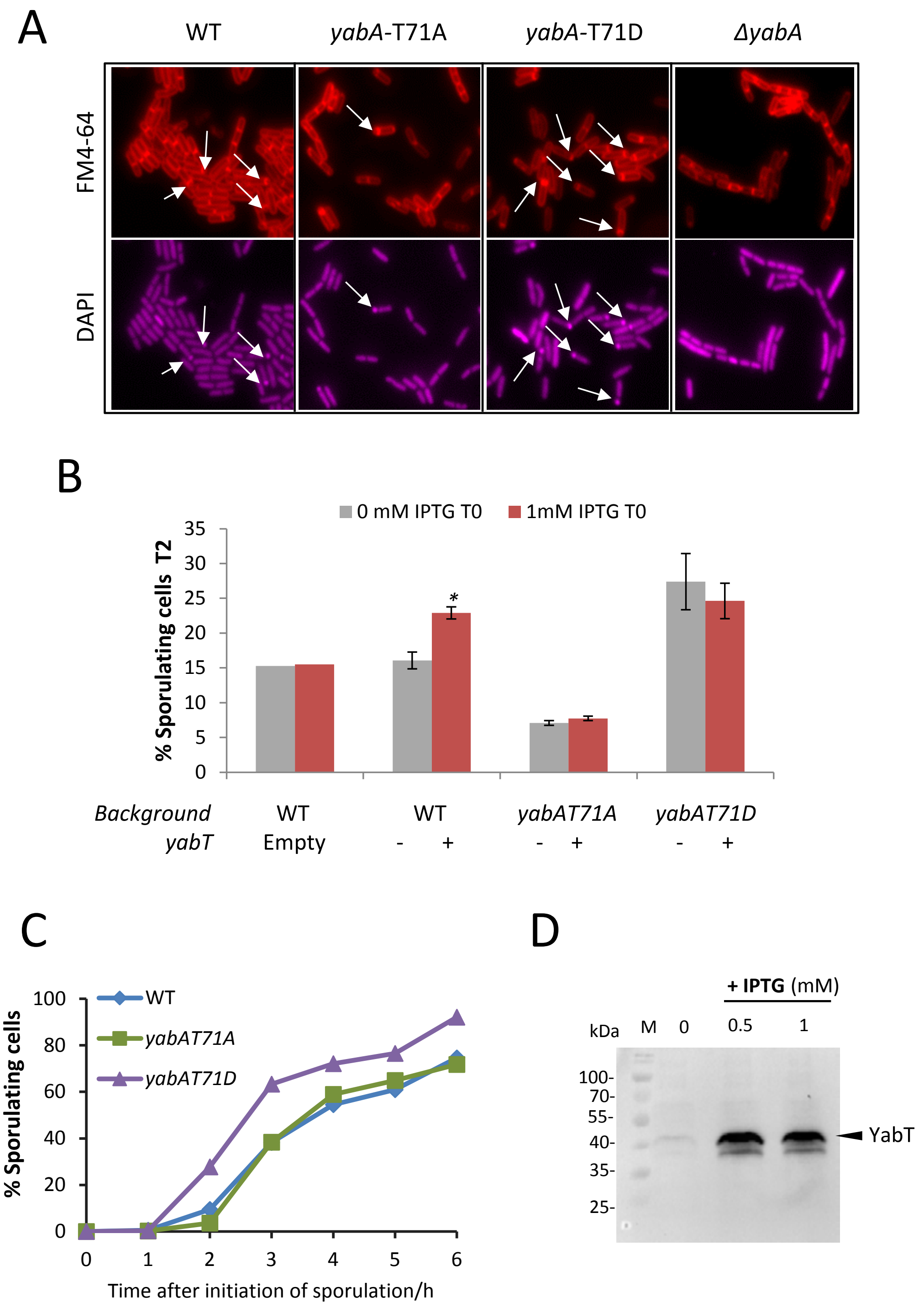

1.6. Supplementary Figure 6.

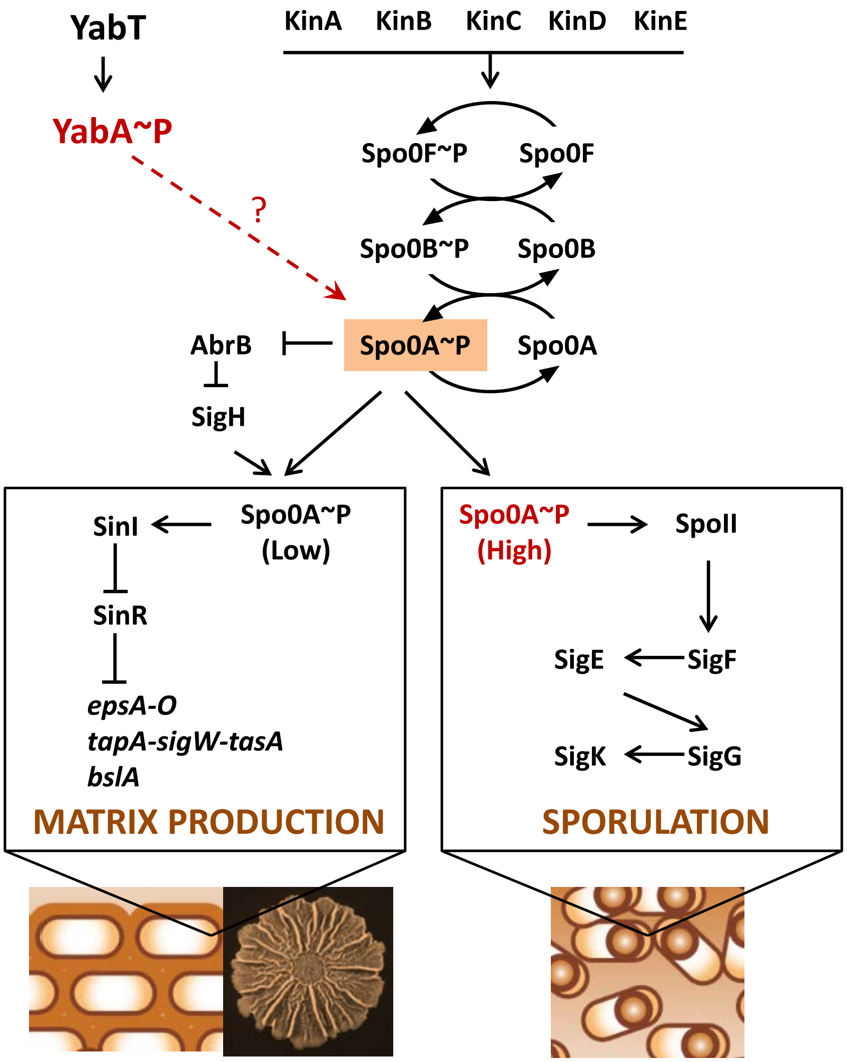

Figure S6. Illustration of the role of YabT-mediated phosphorylation of YabA. The phosphorylation cascade for activation of the master regulator Spo0A and its regulatory loops is represented. Upon phosphorylation YabA-P would modulate the activation of Spo0A by a not yet characterized mechanism (dashed red arrow). The intracellular levels of Spo0A-P is triggering expression of sporulation genes (high) or the anti-repressor *sinI* (intermediate to low) leading to the relief of the SinR-mediated repression of the biofilm matrix gene expression.

2. Supplementary Tables

2-1 Supplementary Table 1: Strain list

A- Strains *B. subtilis*		
**Strains**	**Genotype**	**Source/Construction**
CCBS185	168 *trpC2 Δupp* (Pr-neo)	Laboratory stock, RLC lab.
JJS42	168 *trpC2 Δupp ΔyabA*	Noirot-Gros *et al.,* (2006)
TGG158	168 *trpC2 Δupp yab*AT71A (YabA-Phosphoablative)	This study, see text for details
TGG148	168 *trpC2 Δupp yab*AT71D (YabA-Phosphomimetic)	This study, see text for details
TGG137	168 *trpC2 Δupp ΔyabT::Spec*	This study, see text for details
TGG160	168 *trpC2 Δupp ΔyabT::Spec yab*AT71A	This study, TGG137 x TGG158
TGG161	168 *trpC2 Δupp ΔyabT::Spec yab*AT71D	This study, TGG137 x TGG148
TGG9	168 *trpC2 amyE::Pxyl-gfp-yab*AT71A	This study, CCBS185 x pSG1729 yabAT71A
TGG12	168 *trpC2 amyE::Pxyl-gfp-yab*AT71D	This study, CCBS185 x pSG1729 yabAT71A
TGG36	168 trpC2 Δupp yabAT71A amyE::Pxyl-gfp-yabAT71A	This study, TGG158 x pSG1729 yabAT71A
TGG38	168 trpC2 Δupp yabAT71D amyE::Pxyl-gfp-yabAT71D	This study, TGG148 x pSG1729 yabAT71D
TGG167	168 *trpC2 Δupp* x pDG148-3Flag-yabT	This study, CCBS185 x pDG148-3Flag yabT
TGG169	168 *trpC2 Δupp ΔyabT::Spec* x pDG148-3Flag-yabT	This study, TGG137 x pDG148-3Flag yabT
TGG173	168 *trpC2 Δupp PspoIIA-luc*	This study, CCBS185 x luc fusion spoIIA
TGG174	168 *trpC2 Δupp PspoIIID-luc*	This study, CCBS185 x luc fusion spoIIID
TGG176	168 *trpC2 Δupp yab*AT71A *PspoIIA-luc*	This study, TGG158 x luc fusion spoIIA
TGG177	168 *trpC2 Δupp yab*AT71A *PspoIIID-luc*	This study, TGG158 x luc fusion spoIIID
TGG179	168 *trpC2 Δupp yab*AT71D *PspoIIA-luc*	This study,TGG148 x luc fusion spoIIA
TGG180	168 *trpC2 Δupp yab*AT71D *PspoIIID-luc*	This study, TGG148 x luc fusion spoIIID
TGG182	168 *trpC2 Δupp ΔyabA PspoIIA-luc*	This study, JJS42 x luc fusion spoIIA
TGG183	168 *trpC2 Δupp ΔyabA PspoIIID-luc*	This study, JJS42 x luc fusion spoIIID
TGG188	168 *trpC2 Δupp Pspo0A-gfp*	This study, CCBS185 x pBSBII-spo0A
TGG189	168 *trpC2 Δupp yab*AT71A *Pspo0A-gfp*	This study, TGG158 x pBSBII-spo0A
TGG190	168 *trpC2 Δupp yab*AT71D *Pspo0A-gfp*	This study, TGG148 x pBSBII-spo0A
TGG191	168 *trpC2 Δupp ΔyabA Pspo0A-gfp*	This study, JJS42 x pBSBII-spo0A

**B- Strains *E. coli***		
M15	pREP4-groESL	Amrein *et al.*, (1995)
DH10B	*F- endA1 recA1 galE15 galK16 nupG rpsL ΔlacX74 Φ80lacZΔM15 araD139 Δ(ara,leu)7697 mcrA Δ(mrr-hsdRMS-mcrBC) λ-*	Grant *et al.*, (1990)
ER2566	*F- λ- fhuA2 [lon] ompT lacZ::T7 gene 1 gal sulA11 Δ(mcrC-mrr)114::IS10 R(mcr-73::miniTn10-TetS)2 R(zgb-210::Tn10)(TetS) endA1 [dcm]*	NEB
M15-yabT	M15 qQE-30-yabT	This study, M15 x pQE-30-yabT
ER2566-YabA	ER2566 pSMG251	Laboratory stock
ER2566-YabAT71A	ER2566 pSMG251-YabAT71A	This study, ER2566 x pSMG251-YabAT71A
ER2566-YabAT71D	ER2566 pSMG251-YabAT71D	This study, ER2566 x pSMG251-YabAT71A

**C- Strains *S.cerevisiae***		
PJ69-4α	*MATα trp1-901 leu2-3,112 ura3-52 his3-200 gal4Δ gal180Δ LYS2::GAL1-HIS3 GAL2-ADE2 met2:: GAL7-lacZ*	James *et al.*, (1996)
PJ69-4a	*MATa trp1-901 leu2-3,112 ura3-52 his3-200 gal4Δ gal180Δ LYS2::GAL1-HIS3 GAL2-ADE2 met2:: GAL7-lacZ*	James *et al.*, (1996)
131	PJ69-α, pGAD-C1 : *Leu2 Gal4AD*	Laboratory coll.
305	PJ69-α, pGAD-C1 : *Leu2 Gal4AD-yabA*	Laboratory coll.
5816	PJ69-α, pGAD-C1 : *Leu2 Gal4AD-yabAT71A*	This study
5712	PJ69-α, pGAD-C1 : *Leu2 Gal4AD-yabAT71D*	This study
1534	PJ69-α, pGAD-C1 : *Leu2 Gal4AD-dnaA*	Laboratory coll.
39	PJ69-α, pGAD-C1 : *Leu2 Gal4AD-dnaN*	Laboratory coll.
687	PJ69-α, pGAD-C1 : *Leu2 Gal4AD-acuB*	Laboratory coll.
675	PJ69-α, pGAD-C1 : *Leu2 Gal4AD-tlpA*	Laboratory coll.
121	PJ69-a, pGBDU-C1 : *Ura3 Gal4BD*	Laboratory coll.
319	PJ69-a, pGBDU-C1 : *Ura3 Gal4BD-yabA*	Laboratory coll.
5818	PJ69-a, pGBDU-C1 : *Ura3 Gal4BD-yabAT71A*	This study
5812	PJ69-a, pGBDU-C1 : *Ura3 Gal4BD-yabAT71D*	This study
592	PJ69-a, pGBDU-C1 : *Ura3 Gal4BD-dnaA*	Laboratory coll.
52	PJ69-a, pGBDU-C1 : *Ura3 Gal4BD-dnaN*	Laboratory coll.
3580	PJ69-a, pGBDU-C1 : *Ura3 Gal4BD-acuB*	Laboratory coll.
754	PJ69-a, pGBDU-C1 : *Ura3 Gal4BD-tlpA*	Laboratory coll.

2-2 Supplementary Table 2

Oligonucleotides list:

Oligonucleotides for mutagenesis		
**Nom**	**Sequence 5' to 3'**	**Description**
phleo5'	GAGCTCGAATTCACTGGCCGTCG	K7 phleo - forward - complementary to phleo 5'
phleo3'	CGACCTGCAGGCATGCAAGCT	K7 phleo - reverse - complementary to phleo 3'
yabAT71AF	GAGCTCGAATTCACTGGCCGTCGCAAAAGCACAGAAGACAG AGCAAACTG	*yabA*-forward-mutation T71A
yabAT71AR	CGACCTGCAGGCATGCAAGCTCTGTCTTCTGTGCTTTTGATTC	*yabA*-reverse-mutation T71A
yabAT71DF	GAGCTCGAATTCACTGGCCGTCGCAAAAGACCAGAAGACAG AGCAAACTGATATAG	*yabA*-forward-mutation T71D
yabAT71DR	CGACCTGCAGGCATGCAAGCTCTGTCTTCTGGTCTTTTGATTC TTTTTTATCA	*yabA*-reverse-mutation T71D
YabA Fwd	GTGTCCGCTTTAAGAAAGCG	*yabA* exterior-forward
YabA Rev	ATGCAGATCGTTGGTATACCCG	*yabA* exterior-reverse
**Oligonucleotides for qPCR**		
OriL3-F	CCCAGCATCTTGTAAGGTCAAT	*thdF*- 4212889…4211510 forward
OriL3-R	TTATGTCAGCAACACACGTCAC	*thdF*- 4212889…4211510 reverse
TerL3-F	GGGAGTGGTTGAATGTTCCTA	*proH/yoxE* - 2017711...2016845 fwd
TerL3-R	ATAATAGCGTGTCACCTCATCG	*proH/yoxE* - 2017711...2016845 rev
**Oligonucleotides for cloning**		
yabA-F-Eco	ATCCGGAATTCTTGGATAAAAAAGAGTTATTTGATACAG	*yabA*-forward- EcoRI
yabA-R-SalI	ACGCGTCGACTATTTTTTATTTAAGAATGACAGACAG	*yabA*-reverse-SalI
yabA-apa	ATGGGCCCTTGGATAAAAAAGAGTTATTTGATACAG	*yabA*-forward-ApaI
yabA-NdeI	GGTAAAACATATGGATAAAAAAGAG	*yabA*-reverse-NdeI
YabT-F-SalI	GATGTCGACATGATGAACGACGCTTTGA	*yabT*-forward-SalI
YabT-R-SphI	GCCGCATGCTCAGATTAAGAAAAAGATAATATAGGCG	*yabT*-reverse-SphI
OSMG 464	AATTAATACGACTCACTATAGGG	T7 promoter-forward
OSMG 438	CAAAAAACCCCTCAAGACCC	T7 terminus-reverse
OSMG 538	AGGCTCGAGCTATTTTTTATTTAAGAATGAC	*yabA*-reverse-XhoI
1729-F	TCTAGAAAGGAGATTCCTAGGATGGGTACC	amyE N-terminal fwd
1729-R	GAATTCGATATCAAGCTTATCGATACCGTC	amyE N-terminal rev
pDG148-Bpspac 161	ACATCCAGAACAACCTCTGC	forward
pDG148-Apspac 162	TATGTAAGATTTAAATGCAACCG	reverse

Oligonucleotides for yeast-two-hybrid		
mADA1	CGCGTTTGGAATCACTACAGG	pGAD-forward
mBD1	GGCTTCAGTGGAGACTGATA	pGBDU-forward
mBD2	TCAGAGGTTACATGGCCAAG	pGAD and pGBDU- rev
mAD1ext	AACGGTCCGAACCTCATAAC	pGAD-exterior-fwd
mBD1ext	GTCTCCGTCGACTAGGGCAC	pGBDU-exterior-fwd
mBD2ext	AGCTTCTGAATAAGCCCTCG	pGAD and pGBDU- ext- rev

2-3 Supplementary Table 3

Bacterial and yeast plasmid list:

Bacterial plasmids				
**Plasmids**	**Relevant genotype**	**Resistant**		**source/construction**
		E. coli	***B.subtilis***	
pDG148	*Pspac*	Amp^R	Kn^R $Phleo^R$	Stragier *et al.*, (1988)
pDG148F	*Pspac:3xflag*	Amp^R	Kn^R $Phleo^R$	Laboratory coll.
pDG148-yabT	*Pspac: yabT*	Amp^R	Kn^R $Phleo^R$	This work
pDG148F-yabT	*Pspac:3xflag-yabT*	Amp^R	Kn^R $Phleo^R$	This work
pSG1729	*bla amyE3' spc Pxyl–'gfpmut1 amyE5' - N-ter fusion vector*	Amp^R	Spc^R	Lewis *et al.*, (1999)
pSG1729-yabA	*Pxyl-gfp-yabA*	Amp^R	Spc^R	Noirot-Gros *et al*,. (2006)
pSG1729-yabAT71A	*Pxyl-gfp-yabAT71A*	Amp^R	Spc^R	This work
pSG1729-yabAT71D	*Pxyl-gfp-yabAT71D*	Amp^R	Spc^R	This work
pSMG201	*PT7*	Kn^R		Laboratory stock
pSMG251	*PT7-yabA*	Kn^R		Laboratory stock
pSMG251-T71A	*PT7-yabA*T71A	Kn^R		This work
pSMG251-T71D	*PT7-yabA*T71D	Kn^R		This work
pQE-30-yabT	*PT5-yabT*	Amp^R Cm^R		Jers *et al.*, (2010)
pBSBII-spo0A	*Pspo0A*	Amp^R	Spc^R	Laboratory stock

Yeast plasmids				
**Plasmids**	**Relevant genotype**	**Resistant**		**source/construction**
		E. coli	***S. cerevisiae***	
pGAD-C1	*Gal4AD LEU2 Amp*	Amp^R	L	James *et al*., (1996)
pGBDU-C1	*Gal4BD URA3 Amp*	Amp^R	U	James *et al*., (1996)
pGAD-dnaA	*Gal4AD-dnaA LEU2 Amp*	Amp^R	L	Laboratory coll.
pGBDU-dnaA	*Gal4BD-dnaA URA3 Amp*	Amp^R	U	Laboratory coll.
pGAD-dnaN	*Gal4AD-dnaN LEU2 Amp*	Amp^R	L	Laboratory coll.
pGBDU-dnaN	*Gal4BD-dnaN URA3 Amp*	Amp^R	U	Laboratory coll.
pGAD-yabA	*Gal4AD-yabA LEU2 Amp*	Amp^R	L	Laboratory coll.
pGBDU-yabA	*Gal4BD-yabA URA3 Amp*	Amp^R	U	Laboratory coll.
pGAD-yabAT71A	*Gal4AD-yabAT71A LEU2 Amp*	Amp^R	L	This work
pGBDU-yabAT71A	*Gal4BD-yabAT71A URA3 Amp*	Amp^R	U	This work
pGAD-yabAT71D	*Gal4AD-yabAT71D LEU2 Amp*	Amp^R	L	This work
pGBDU-yabAT71D	*Gal4BD-yabAT71D URA3 Amp*	Amp^R	U	This work
